# Visual saltation illusion induced by flashes of subjective contours

**DOI:** 10.1177/20416695231191241

**Published:** 2023-08-11

**Authors:** Hiroyuki Ito, Keiya Kubo, Sheryl Anne Manaligod de Jesus

**Affiliations:** 12923 Kyushu University, Japan; 12923 Kyushu University, Japan; 12923 Kyushu University, Japan;; 5305 University of Guam, USA

**Keywords:** visual saltation illusion, Kanizsa triangle, subjective contour

## Abstract

The visual saltation illusion of a Kanizsa-type subjective triangle was demonstrated. After a subjective/real triangle was flashed twice at the same position, another subjective/real triangle was flashed at a displaced position. In a typical case, the second flash was perceived to occur midway between the first and third flash positions. This study showed that the rated illusion strength for the Kanizsa and real triangles largely depended on stimulus onset asynchrony and retinal eccentricity and that the illusion rating was the same between the Kanizsa and real gray triangles when they were presented on black disks (or inducers). When the real triangle was presented in isolation, the illusion rating was lower. Presenting flashes on disks appears to enhance the saltation illusion for both the Kanizsa and real triangles possibly due to a stronger crowding effect or shape changes of inducers enhancing the perception of object appearance and disappearance. Various types of saltation illusions with a Kanizsa triangle are demonstrated in a video.

Geldard and Sherrick (1972) found that multiple taps in rapid succession presented on separate skin positions cause a “hopping” sensation regularly in time and space between the stimulated skin positions (cutaneous rabbit illusion or saltation illusion). Similar illusions are known to occur for vision and audition (Bremer et al., 1977; Geldard, 1975). A vision version of the reduced-rabbit illusion (Geldard, 1975) has been used to test a visual saltation illusion (e.g., Geldard (1976) and Khuu et al. (2011)), that is, a visual object was flashed at a position twice, followed by another object flashes at a distant position. Although the second flash occurs at the same position as the first one, the second flash is perceived to occur midway between the first and third flash positions (see Figure 1) when spatiotemporal conditions are appropriate.

**Figure 1.**
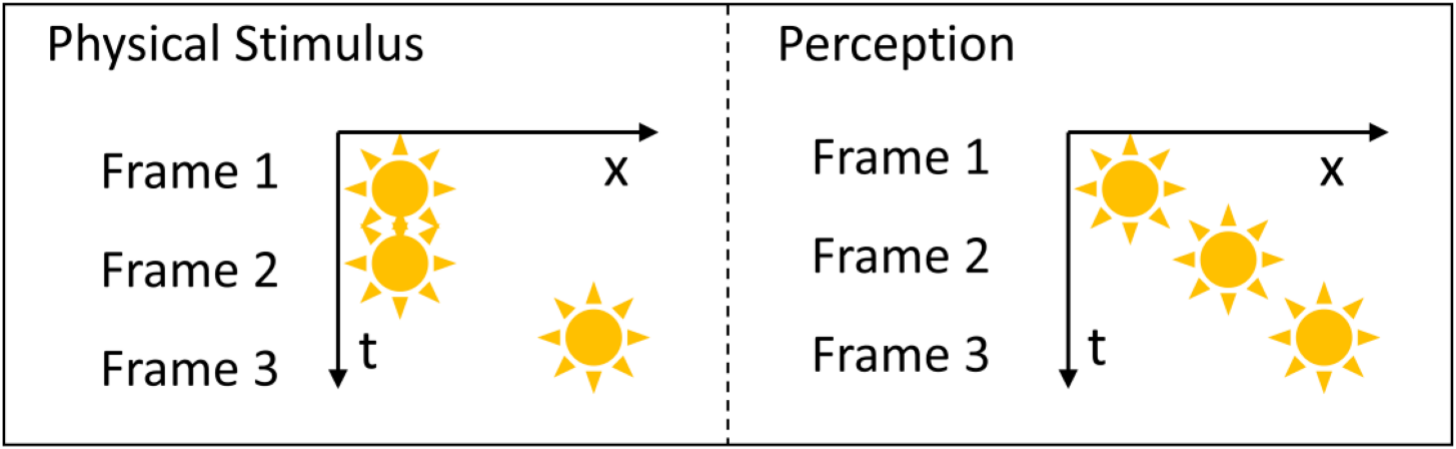
Reduced-rabbit paradigm of the visual saltation illusion.

In this report, the visual saltation illusion with a Kanizsa-type subjective triangle (Kanizsa, 1955) was demonstrated through two experiments and a video presentation. It is known that a Kanizsa-type subjective figure produces apparent motion similarly to a real figure (Mather, 1988; Ohmura, 1981; Ramachandran, 1985). This report aims to demonstrate the visual saltation illusion with the Kanizsa triangle as a counter part of the research on apparent motion of the Kanizsa-type subjective triangle. In Experiment 1, the visual saltation illusion with real and subjective triangles were compared varying temporal conditions to search for an optimum temporal condition for the visual saltation illusion of both triangles. Experiment 2 tested the effect of retinal eccentricity of stimulus presentation and the effect of inducers of the Kanizsa triangle on the saltation illusion from a point of view of visual crowding. Finally, we provided a video that could demonstrate the possibilities of various kinds of saltation illusions with the Kanizsa triangle.

## Experiment 1

### Method

#### Observers

Fourteen undergraduate or graduate students participated in Experiment 1. This study has been approved by the local ethics committee of Kyushu University.

#### Apparatus and Stimuli

**Figure 2.**
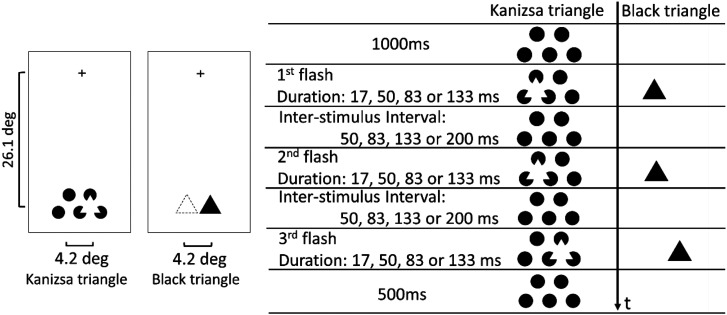
Spatial and temporal configurations of a stimulus presentation.

A 17-inch organic light-emitting diode display (Sony, PVM-A170) was used to present flashing stimuli. The screen was refreshed at 60 Hz. The whole display was rotated at a 90° orientation to attain the large vertical eccentricity of stimulus presentation. The viewing distance was 60 cm. Figure 2 shows the spatial and temporal configurations of the stimuli. The first and second flashes occurred at the same position and the third flash occurred at a horizontally distant position. The eccentricity of the stimulus presentation was 26.1°. One “side” of the Kanizsa triangle was 4.2° and the diameter of the black disks as inducers was 2.6°. A black triangle corresponding to the Kanizsa triangle in size and presented positions were used for comparison. Luminance of the white or black part was 101.5 or 0.17 cd/m^2^, respectively. The duration of a flash and the inter-stimulus interval (ISI) between flashes were varied as described in the figure.

#### Procedure

**Figure 3.**
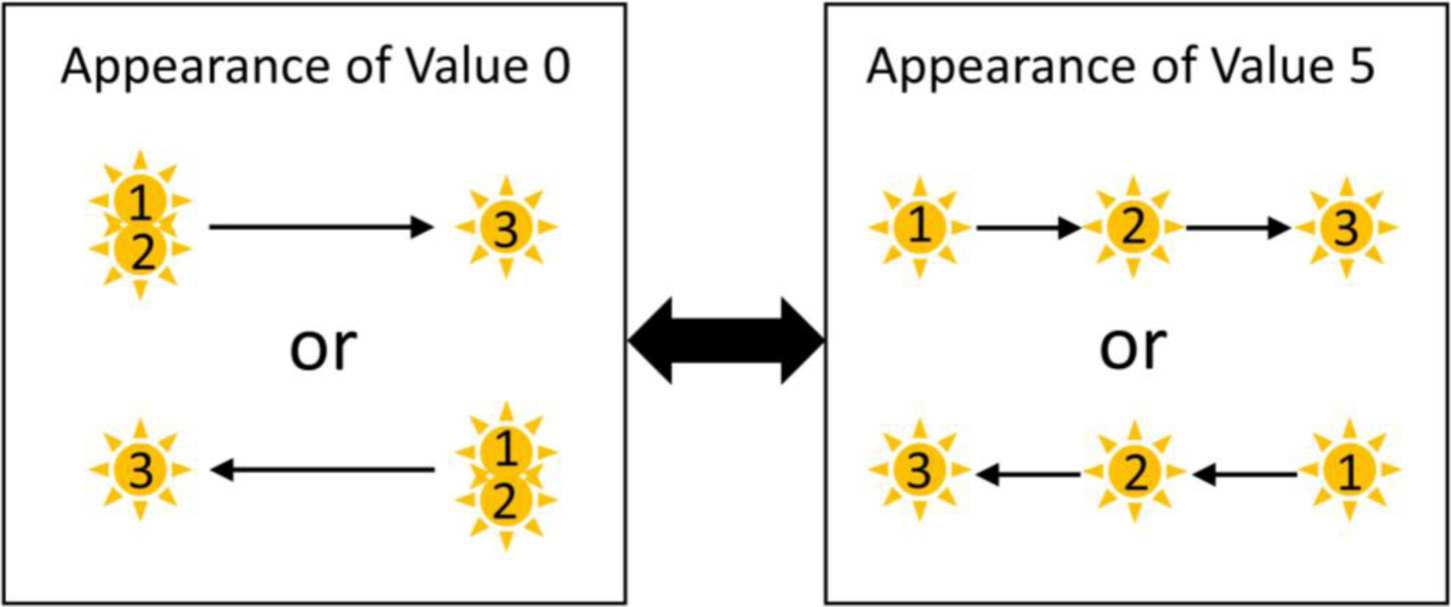
Rating method with a six-degree scale. When the second flash was seen at the physically flashed position, the value was 0 (Left panel). When the second flash was seen midway between the first and third positions, the value was 5 (Right panel).

Observers performed subjective evaluations of the illusory effect. The concept of the visual saltation illusion was explained to the observers before the experiment. They judged the illusory effect using a six-degree scale (0–5) shown in Figure 3. The direction of flash-position shift was randomly assigned as left-to-right or right-to-left. In one trial, the stimulus sequence was repeated three times before the evaluation was performed. There were three factors, two stimulus types (i.e., Kanizsa or black triangles), four levels of ISIs, and four levels of flash durations. One session included 32 conditions, presented in a random order. After one training session, four sessions were conducted.

### Results and Discussion

**Figure 4.**
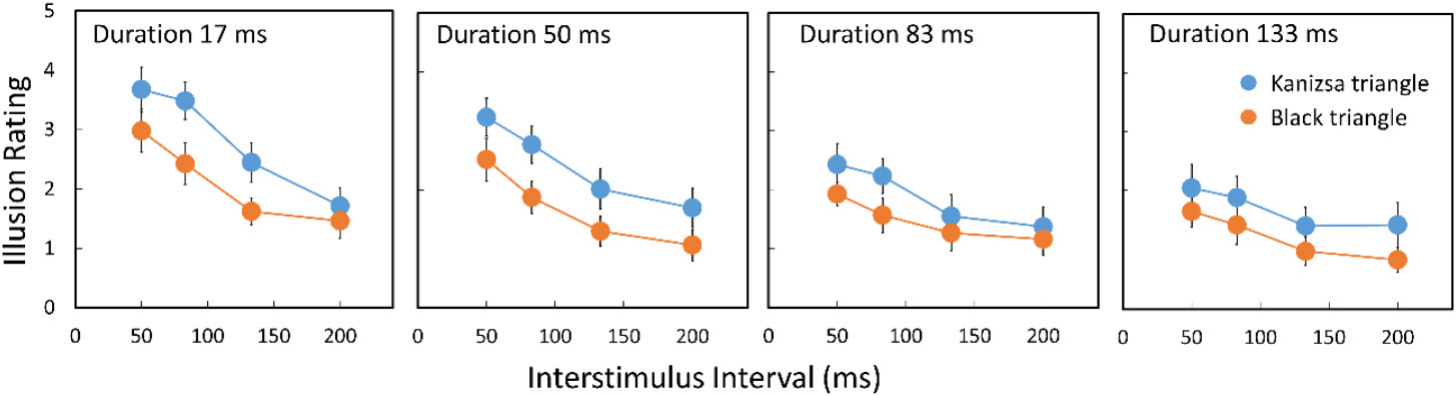
The results in Experiment 1. Error bars indicate standard errors.

**Figure 5.**
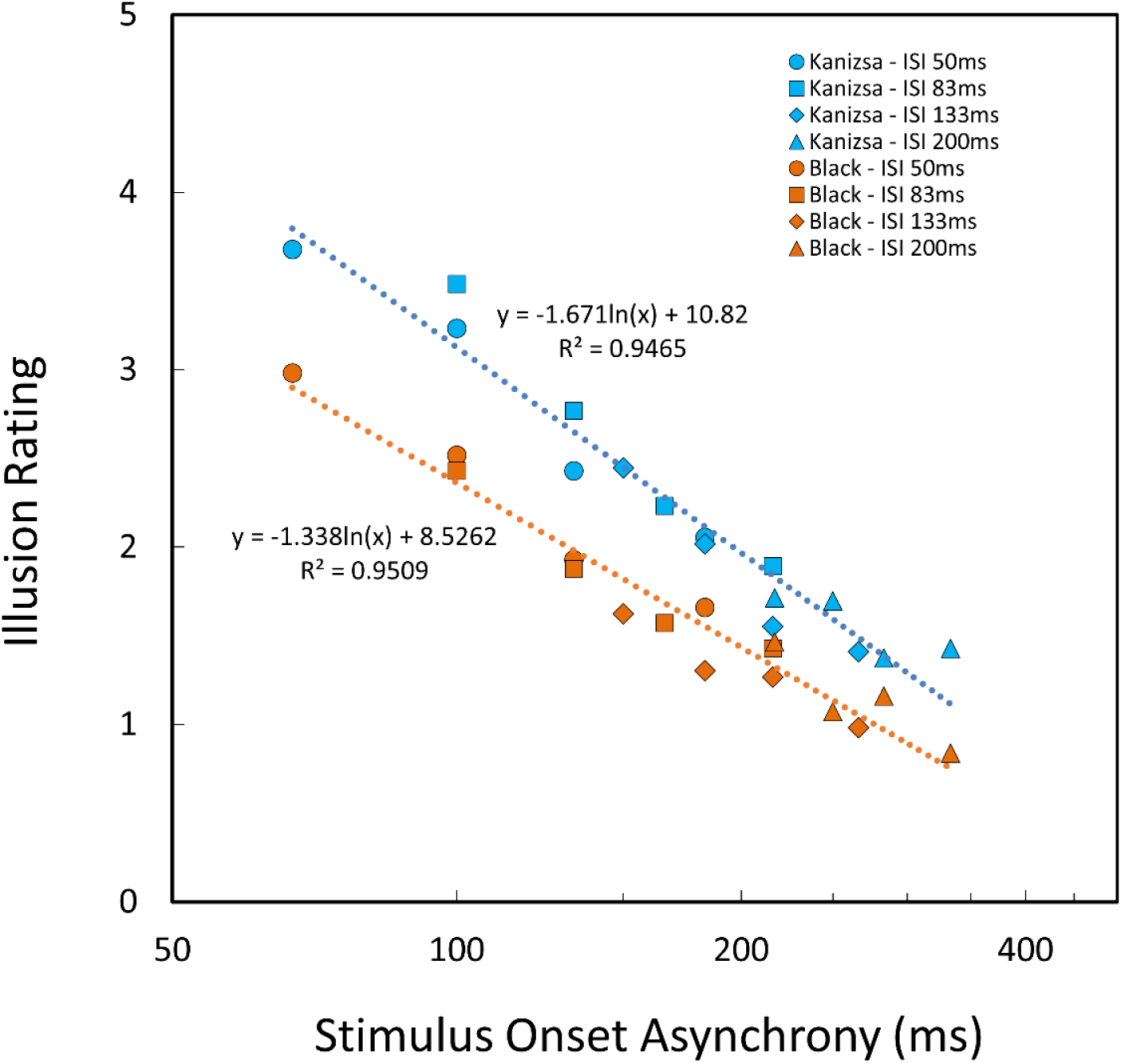
Illusion ratings as a function of SOA. Please note that the horizontal axis uses a log scale.

*Note.* SOA = stimulus onset asynchrony.

The ratings of the illusory effect under the Kanizsa triangle condition were higher than those of the black triangle, *F*(1, 13) = 9.754, *p *= .0081, *ηp^2^^ ^* = 0.4287. The ratings for the Kanizsa and black triangles significantly decreased with the increase in duration, *F*(3, 39) = 9.399, *p *= .0001, *ηp^2^^ ^* = 0.4196 and ISI, *F*(3, 39) = 19.256, *p *< .0001, *ηp^2^^ ^* = 0.5970. Although the interaction between duration and ISI was also found to be significant, the effect size was small, *F*(9, 117) = 2.347, *p *= .0180, *ηp^2^^ ^* = 0.1529. The results shown in Figure 4 could be summarized by replotting the rating data as a function of stimulus onset asynchrony (SOA) as shown in Figure 5. In our stimulus display, the second flash appeared at least 50 ms after the first flash disappearance (i.e., at least 50 ms ISI). Thus, the time interval between the first and the second flash onsets is equal to the duration of the first flash plus ISI between the first and second flashes, as shown in Figure 2. This is also the case for the second and the third flashes. In both Kanizsa and black triangle conditions, the ratings linearly decreased with the log SOA. The *R^2^* was quite large (0.95, *p *< .0001) for both triangles. The illusory effect under the Kanizsa triangle condition was always rated as higher than that under the black triangle condition when the SOA was the same. Geldard (1976) varied ISI between the second and third flashes and found that the ISI was a determinant of the saltation effect. However, our results may show that SOA is more crucial than ISI within the range we have tested, indicating the importance of onset signals of the object appearance for the visual saltation illusion. In apparent motion research employing two flash stimuli, SOA has been known as a critical factor (e.g., onset-onset law, Kahneman, 1967). This might suggest that the visual saltation illusion and apparent motion share a common low-level process.

We thought that the Kanizsa triangle needed more time to be processed than a real triangle did. Thus, we expected that the shorter durations and ISIs were favored by luminance-defined objects, resulting in a stronger illusion of a black triangle under the smaller SOA conditions. We widely varied the time parameters in Experiment 1, to confirm two evaluation peaks in a time scale that was different between subjective and real triangles. However, within the range of temporal conditions in durations and ISIs (thus SOAs) tested here, the Kanizsa triangle always produced a stronger effect than the black triangle and both types of triangles showed the strongest saltation illusion at the smallest SOA condition. Therefore, we reject the hypothesis of the processing time difference.

Although the appearance of the Kanizsa triangle decreased the black area of the inducers, the amount of the change in luminance was less than the conditions when the black triangle appeared. In addition, the Kanizsa triangle dropped in visibility at a large eccentricity although the black figures did not. Nevertheless, the illusory effect under the Kanizsa triangle condition was larger than that under the black triangle condition.

## Experiment 2

Experiment 1 demonstrated that the Kanizsa triangle could produce the visual saltation illusion. However, the reason for the higher evaluation of the Kanizsa triangle condition under a wide variety of temporal conditions was not clear. One reviewer of this article suggested that visual crowding (Whitney & Levi, 2011, for review) caused by the inducers constituting the Kanizsa triangle (black disks or “Pac-Men”) might affect the strength of the illusion. While the black triangle used in Experiment 1 was spatially isolated (i.e., no flanker object around it), the Kanizsa triangle was necessarily produced by the flankers (i.e., inducers). It is possible that the flashed positions became perceptually ambiguous due to the flankers, particularly when viewed in far peripheral vision. In Experiment 2, we investigated the effect of the inducers (or flankers) on the visual saltation illusion under the Kanizsa and real triangle conditions.

We also varied the retinal eccentricity of the stimulus presentation. The visual saltation illusion with light-emitting-diode (LED) flashes was shown not to arise in central vision but to be evident in far peripheral vision (Geldard, 1976; Lockhead et al., 1980). As our apparatus and stimulus configuration were completely different from Geldard (1976), the effect of eccentricity should be confirmed. In addition, if the cause of the greater illusory effect under the Kanizsa triangle condition in Experiment 1 is that the presented triangle was subjective, a smaller eccentricity might produce a thoroughly greater effect under the Kanizsa triangle condition than the real triangle condition because the Kanizsa triangle may be more apparent at a smaller retinal eccentricity.

### Method

#### Observers

Nine graduate students including the third author and two staff members of Kyushu University including the first author participated in Experiment 2.

#### Apparatus and Stimuli

**Figure 6.**
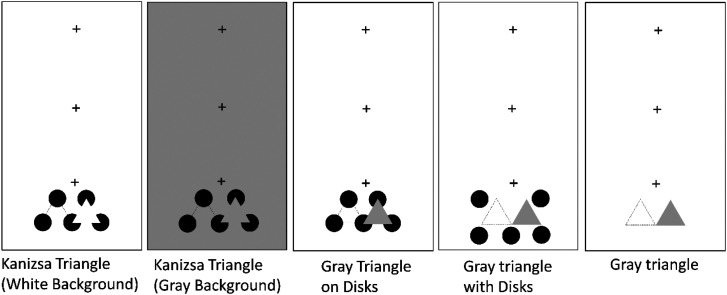
Tested conditions in Experiment 2. The triangles with broken lines indicate the first and second triangle presentation positions. The fixation cross was presented at one of the three positions to vary the retinal eccentricity of the stimulus presentation.

The apparatus was the same as in Experiment 1. Figure 6 shows the stimuli used in Experiment 2. The following five spatial configurations were compared.

*Kanizsa Triangle (White Background)*: This configuration was the same as Experiment 1.

*Kanizsa Triangle (Gray Background)*: This was the Kanizsa triangle display with a darker background (39.1 cd/m^2^). The luminance of the Kanizsa triangle region was the same as that in other gray triangle conditions.

*Gray Triangle on Disks*: In this configuration, a gray triangle was presented in place of the Kanizsa triangle. The luminance of the gray triangle was 39.1 cd/m^2^. The gray triangle partly occluded the black disks when it appeared.

*Gray Triangle with Disks*: The flankers for the gray triangle were five black disks. The size of the disks was the same as that of the inducers of the Kanizsa triangle. The gray triangle never occluded the black disks. Thus, there was no change in shape and luminance in the disk regions even when the gray triangle appeared.

*Gray Triangle*: This configuration did not include black disks*.* The gray triangle was spatially isolated as used in the black triangle in Experiment 1.

The elements were very crowded in the *Kanizsa-triangle with a white/gray background* condition and the *gray-triangle-on-disks* condition. Under the *gray-triangle-with-disks* condition, although there were five black disks as flankers, the crowding effect might be a little less than the above-noted three conditions. Additionally, under the *gray-triangle-with-disks* condition, no occlusion occurred when the triangle appeared. The *gray-triangle* condition did not include flankers.

**Figure 7.**
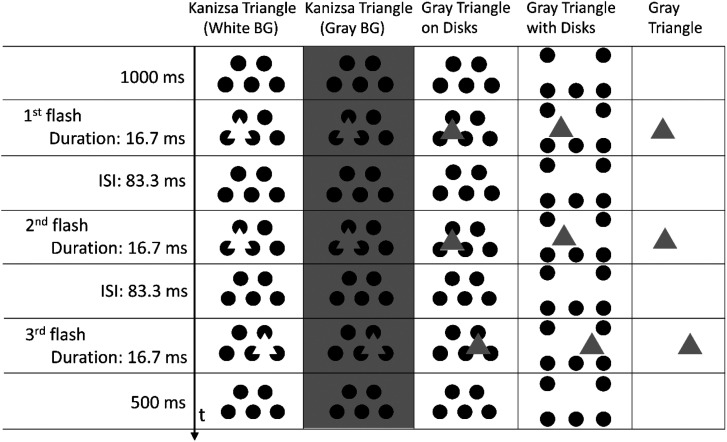
Temporal configuration of stimulus presentation in Experiment 2.

The retinal eccentricity of the stimulus presentation was varied (4.4, 15.8, or 26.1°) by changing the position of the fixation cross as shown in Figure 6. The sizes of the disks and triangles were the same as in Experiment 1. The duration of flashes and the ISI between flashes were 16.7 ms (one frame) and 83.3 ms (five frames), respectively (Figure 7).

#### Procedure

There were two factors, five types of stimulus configurations and three levels of retinal eccentricity as shown in Figure 6. In one session, there were 30 trials (Five Stimulus Configurations × Three Eccentricities × Two Directions (left-to-right and right-to-left directions)) presented in random order. Following a training session, four sessions were conducted. Data from both direction displays were collapsed.

### Results and Discussion

**Figure 8.**
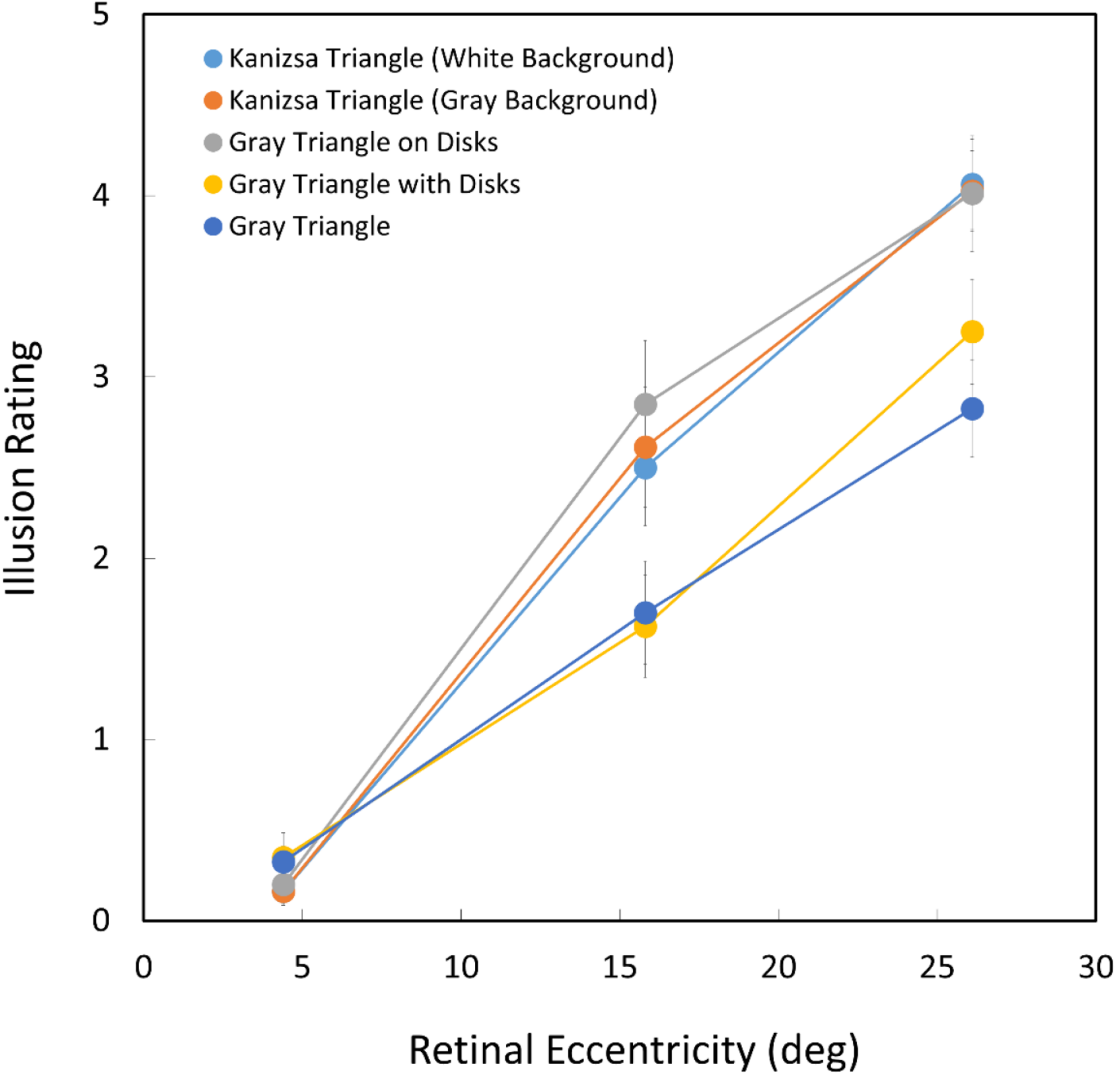
Illusion ratings as a function of retinal eccentricity. Error bars indicate standard errors.

 Figure 8 displays the results of Experiment 2. An analysis of variance was conducted. The data from one observer was removed from the analysis because significant outliers were found in two conditions (the Smirnov–Grubbs’ outlier test, *p < .05*) as noted later. Under all configuration conditions, the retinal eccentricity was a decisive factor, *F*(2, 18) = 239.491, *p *< .0001, *ηp^2^^ ^* = 0.9638, to produce the visual saltation illusion. The effect of stimulus configuration was significant, *F*(4, 36) = 5.203, *p *= .0021, *ηp^2^^ ^* = 0.3663. The interaction between the two factors was also significant, *F*(8, 72) = 5.729, *p *< .0001, *ηp^2^^ ^* = 0.3890. The simple main effect of eccentricity was significant for all stimulus configuration conditions (*p* < .0001). The simple main effect of the five stimulus configuration conditions was little under the smallest eccentricity (4.4°) condition (*p* > .05) because the illusion did not seem to occur. However, under the 15.8° and 26.1° conditions, the simple main effect of stimulus configuration was significant (*p* < .0001). The results of multiple comparisons were the same under the 15.8° and 26.1° eccentricity conditions. The *Kanizsa-triangle (white/gray-background)* conditions and the *gray-triangle-on-disks* condition were highly evaluated without significant difference between the three (*p* > .05). This indicates that the saltation illusion was similarly strong no matter whether the presented triangle was real or subjective when it was presented on disks. The *gray-triangle* condition and the *gray-triangle-with-disks* condition produced a weaker saltation illusion without a significant rating difference between the two (*p* > .05). The difference in rating between any pair from the former three and the latter two stimulus configuration conditions was significantly different (*p* < .05).

Under the former three and the *gray-triangle-with-disks* conditions, there were five disks as flankers. However, the illusion ratings under the *gray-triangle-with-disks* condition were similar to those under the *gray-triangle* condition with no disk. One possible reason is that the crowding effect under the *gray-triangle-with-disks* condition was not strong enough to enhance the illusory effect because they were not as crowded as those under the *Kanizsa-triangle* conditions. As noted earlier, the data from one observer was removed from the statistical analysis through the outlier test. She did not perceive any saltation illusion under the *Kanizsa-triangle (white/gray-background)* conditions and the *gray-triangle-on-disks* condition. She reported that the stimuli under those conditions were perceived as one flash that crashed spatially and temporally. This may suggest that the *Kanizsa-triangle* configuration reduced the spatial resolution through a strong crowding effect and also reduced the temporal resolution of the flashed objects. The reason for temporal resolution reduction is not clear at present. This episode may suggest that the effect of the *Kanizsa-triangle* configuration (not including the *Gray-triangle-with-disks* condition) was strong enough to change the perception of flashes.

Another possible reason is that the disks were not occluded by the gray triangle when they appeared under the *gray-triangle-with-disks* condition. The main difference in stimulus configuration between the former three and the latter two conditions was whether the triangle appeared on disks (i.e., whether the triangle occluded the disks) or not. The shapes of the inducers were disks during ISIs or Pac-Men during triangle presentation no matter whether the triangle was subjective or gray. This change in shape and luminance may indicate ISIs more clearly, disturbing visual persistence or afterimage of the first flash, and enhance the onset of the second flash of the triangle.

As for the *gray-triangle* condition, the rating of the saltation illusion was lower than that under the *Kanizsa-triangle* conditions as found in the black triangle condition in Experiment 1. There was no crowding and no dynamic occlusion in the *gray-triangle* condition. Inversely, the rating under the *gray-triangle-on-disks* condition was almost the same as that under *Kanizsa-triangle (white/gray-background)* conditions with crowding and dynamic occlusion. Thus, the apparently stronger illusory effect under the *Kanizsa-triangle* condition found in Experiment 1 could be explained by crowding or dynamic occlusion. It is not reasonable to conclude the reason for the relatively stronger illusion under the *Kanizsa-triangle* condition than the real triangle condition in Experiment 1 was the subjectivity of the Kanizsa triangle. Please note that we are not suggesting that the subjective contour did not produce the effect. Contrarily, we demonstrated here that the visual saltation illusion is perceived for a Kanizsa-type subjective triangle just as perceived for a real triangle.

From the results of Experiment 1, one could question if the difference in illusory effect between the Kanizsa and real triangles was caused by brightness/contrast differences because the Kanizsa triangle was white while the real triangle was black presented on a white background. We could reject this hypothesis through comparisons of conditions in Experiment 2 that employed gray triangles in five conditions. First, there was a clear difference in illusion evaluation between the *gray-triangle-on-disks* condition and the *gray-triangle* condition. Both gray triangles were presented on a white background. Secondly, there was little difference between the *gray-triangle-on-disks* condition and the *Kanizsa-triangle (white background)* condition. Although a white background was used for both conditions, the triangle color was different, that is, gray or white. Finally, there was little difference between the *Kanizsa-triangle (gray background)* condition and the *gray-triangle-on-disks* condition. In both conditions, gray triangles were presented while the background luminance was different, that is, gray or white. From these comparisons, it does not appear that the brightness/contrast was a major factor to determine the saltation illusion in Experiments 1 and 2.

To summarize our speculation on why the Kanizsa configuration is better at creating the saltation effect than the real triangle, (1) a stronger crowding effect resulted in increasing spatial ambiguity, and/or (2) shape (and luminance) changes in inducers enhanced object appearance and disappearance.

## General Discussion

Through two experiments, we showed that the Kanizsa-type subjective figures could produce the visual saltation illusion. In Experiment 1, we found that the ratings of the saltation illusion strongly depended on the SOA and that the ratings of the visual saltation illusion under the Kanizsa triangle condition were higher than that under the black triangle condition within a range of temporal conditions we have tested. Experiment 2 investigated the effect of retinal eccentricity at which the stimulus was presented and the contribution of the inducers of the Kanizsa triangle (black disks or Pac-Men) that could produce a crowding effect as flankers. The results showed that the saltation illusion strongly depended on retinal eccentricity irrespective of the stimulus configuration and suggested that the strength of the illusion was affected by the inducers themselves. The saltation effect for the gray triangle was equally strong to that for the Kanizsa triangle when the gray triangle was presented on the black disks just in place of the Kanizsa triangle. The higher ratings in the Kanizsa triangle condition in Experiment 1 may be explained by the crowding effect or dynamic occlusion (accompanying the shape change in disks). We conclude that the visual saltation illusion could arise for the Kanizsa triangle similarly for a real triangle without distinction and that the existence of inducers (black disks) could enhance the saltation illusion. The visual saltation illusion of the Kanizsa triangle could be a counterpart of the apparent motion of a Kanizsa triangle (Mather, 1988; Ohmura, 1981; Ramachandran, 1985). Both the visual saltation illusion and apparent motion seem to have a nature of postdiction (Eagleman & Sejnowski, 2000; Shimojo, 2013, for a review) in that the latter flash determines the interpretation of the previous event. To carefully compare the two phenomena may be a promising direction of research.

It is possible to present other types of saltation illusions with Kanizsa triangles on the analogy of classical long-range apparent motion, as shown in Video 1. This video demonstrates that the Kanizsa triangle in the visual saltation illusion is not just three corners with luminance changes but, at least perceptually, an object that could be transformed as observed in long-range apparent motion. The *translation* is the stimulus type used in Experiments 1 and 2. In *magnification*, the triangle in the second frame is perceived as the one in an intermediate size and position between those of the triangles in the first and third frames. In *2D rotation*, the triangle in the second frame is perceived as one in an intermediate size, position, and orientation. The saltation illusion also occurs between a combination of the flashes of *subjective and real triangles*. This may suggest that there is no distinction between Kanizsa and real triangles for the occurrence of the visual saltation illusion.

**Video 1.**
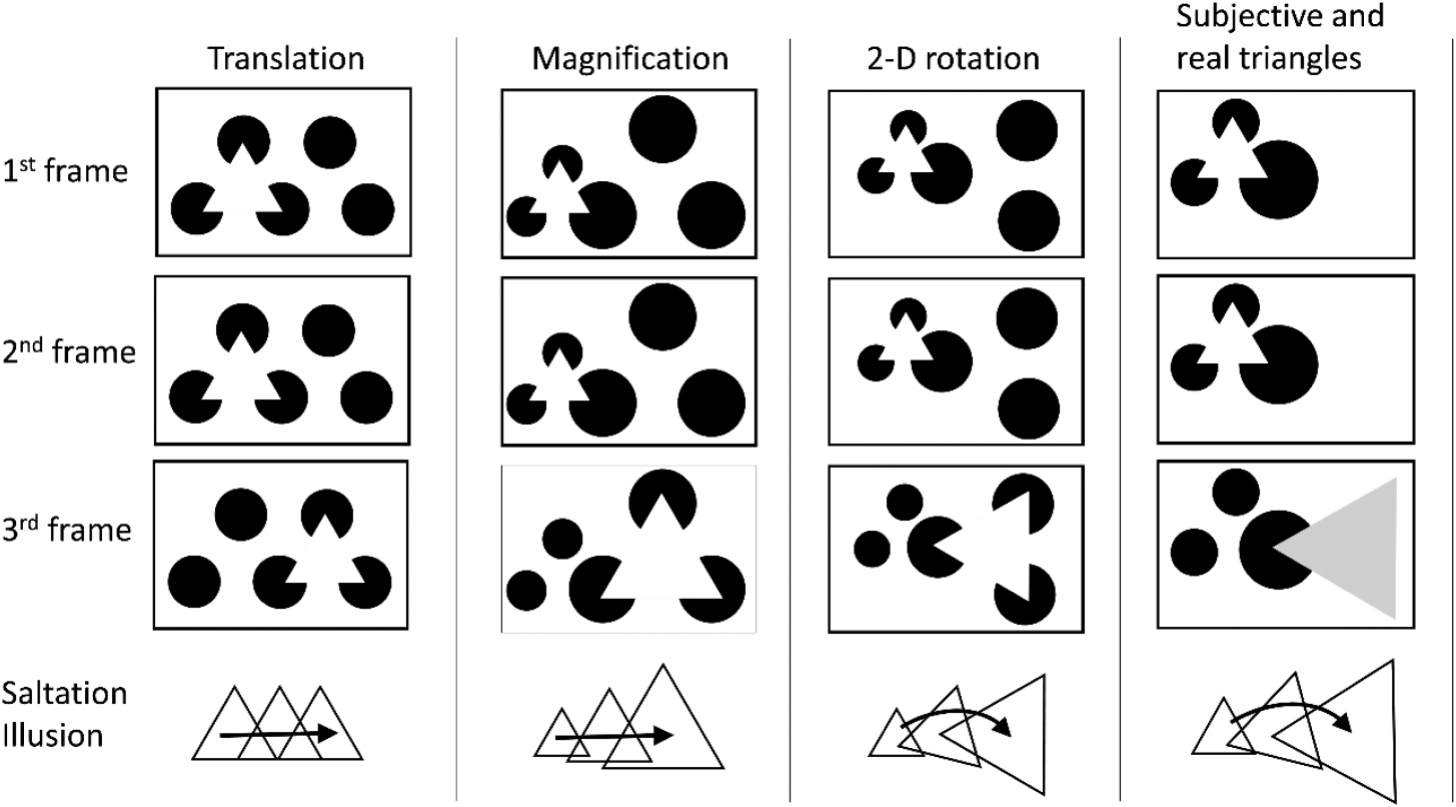
Various types of saltation illusions with Kanizsa triangles. Please view an attached video demonstration (“Saltation_KanizsaT.mp4”).

The object size perception is influenced by the size of the object presented before its presentation (Kawabe, 2008) and by the size of the object presented after its presentation (Kawabe, 2011). There was also research on postdictive change in orientation perception (Kawabe, 2012). As these experiments were conducted in a two-frame apparent motion paradigm, not the same as ours, the demonstration in Video 1 could be tested with the two-frame apparent motion paradigm.

The limitations of this paper should also be noted. First is the problem of the evaluation method. In our evaluation method used here, the “best” illusion was assigned as five, meaning the second flash was perceived at the midway of the first and third flashes. One could ask whether the perceived position of the second flash exceeded the halfway point and appeared closer to the third flash position. Geldard (1976) tested the illusory effect within the range of 15°–40° eccentricity and varied ISIs between the second and third LED flashes with a constant ISI between the first and second flashes. He found that only when the ISI between the second and third flashes was shorter than 50 ms or when the stimulus presentation eccentricity was more than 30° from the fovea, the perceived second flash leap could exceed the 50% relative position from the first flash position and move closer to the third flash position. These spatial and temporal conditions were beyond our stimulus settings. We have seldom observed such cases in our stimuli. To note, only one participant reported that the second flash appeared at a little near to the third flash in a few trials and that she evaluated the effect as “five.” When more comprehensive research is conducted on the visual saltation illusion, the evaluation method should be improved.

The second is the limited stimulus properties tested here, for example, the size of the triangles, distance between the first and the second flash positions, luminance contrast of the inducers, and contrast polarity of the stimulus configuration. We think each stimulus property could modulate the saltation illusion. If the saltation illusion shares a basic mechanism with apparent motion, the stimulus property that could influence apparent motion also could affect the saltation illusion. These properties should be tested in comparison with apparent motion.

The third limitation is the limited object types tested. Although we have investigated the saltation illusion with the Kanizsa triangle, it may be possible that other types of visual objects cause the saltation illusion. In fact, we have observed the saltation illusions with the Ehrenstein illusion figure and the texture-defined square (Ito, 2019). As Khuu et al. (2010) showed that cyclopean objects could cause the saltation illusion, various kinds of second-order stimuli may produce the saltation illusion. Finally, as shown in Video 2, the saltation illusion seems to occur even when the object does not have a definite shape. This movie was provided by Stuart Anstis, a signed reviewer of this article, and suggests a possible research direction for future research.

**Video 2.**
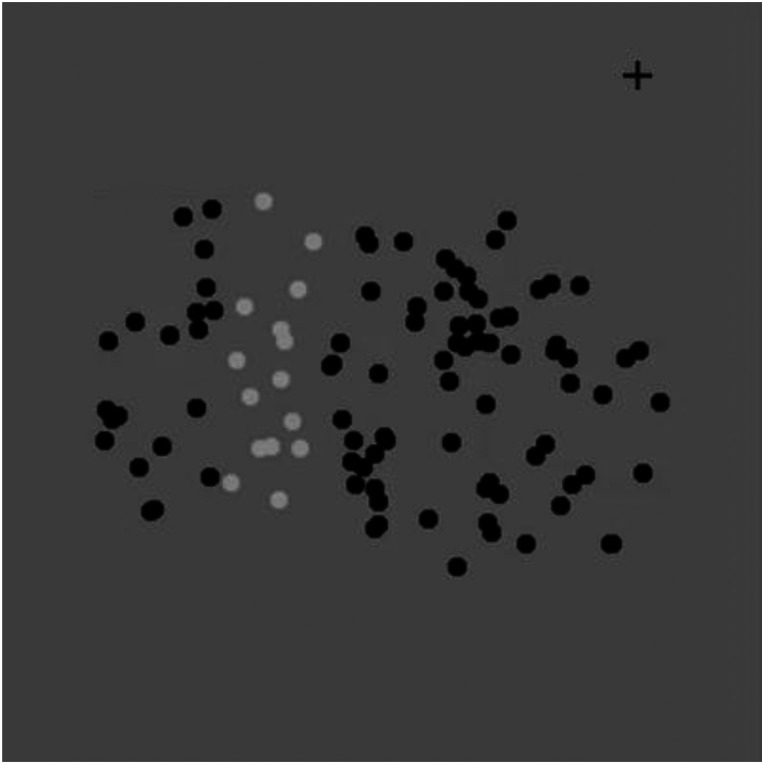
A saltation illusion of an “object” without definite shape (see attached Movie demonstration “Saltation_Anstis.mp4”), produced by Stuart Anstis, a signed reviewer.

In summary, we found in this study that a Kanizsa-type subjective triangle could produce the saltation illusion in the exact same manner as a real triangle. The illusory effect for the Kanizsa triangle was enhanced possibly due to the stimulus configuration rather than its subjectivity. Various types of saltation illusions with Kanizsa triangles (translation, magnification, and rotation) were demonstrated in a video.

## References

[bibr1-20416695231191241] Bremer C. D. Pittenger J. B. Warren R. Jenkins J. J. (1977). An illusion of auditory saltation similar to the cutaneous “rabbit”. American Journal of Psychology, 90, 645–654. 10.2307/1421738 610449

[bibr2-20416695231191241] Eagleman D. M. Sejnowski T. J. (2000). Motion integration and postdiction in visual awareness. Science, 287(5460), 2036–2038. 10.1126/science.287.5460.2036 10720334

[bibr3-20416695231191241] Geldard F. A. (1975). Sensory saltation: Metastability in the perceptual world. Lawrence Erlbaum Associates.

[bibr4-20416695231191241] Geldard F. A. (1976). The saltatory effect in vision. Sensory Processes, 1(1), 77–86. PMID: 1029079. 1029079

[bibr5-20416695231191241] Geldard F. A. Sherrick C. E. (1972). The cutaneous rabbit: A perceptual illusion. Science, 178(4057), 178–179. 10.1126/science.178.4057.178 5076909

[bibr6-20416695231191241] Ito H. (2019). A visual saltation illusion with subjective contours. Perception, 48(1_supplement), 87. (abstract of 41st European Conference on Visual Perception (ECVP) 2018 Trieste). 10.1177/0301006618824879 PMC10422910 37575682

[bibr7-20416695231191241] Kahneman D. (1967). An onset-onset law for one case of apparent motion and metacontrast. Perception & Psychophysics, 2, 577–584. 10.3758/BF03210272

[bibr8-20416695231191241] Kanizsa G. (1955). Margini quasi-percettivi in campi con stimolazione omogenea. Rivista di Psicologia, 49(1), 7–30. CRID: 1570854174304201088.

[bibr9-20416695231191241] Kawabe T. (2008). Spatiotemporal feature attribution for the perception of visual size. Journal of Vision, 8(8), 7–7. 10.1167/8.8.7 18831630

[bibr10-20416695231191241] Kawabe T. (2011). Nonretinotopic processing is related to postdictive size modulation in apparent motion. Attention, Perception, & Psychophysics, 73, 1522–1531. 10.3758/s13414-011-0128-4 21472506

[bibr11-20416695231191241] Kawabe T. (2012). Postdictive modulation of visual orientation. PLoS One, 7(2), e32608. 10.1371/journal.pone.0032608 22393421 PMC3290577

[bibr12-20416695231191241] Khuu S. K. Kidd J. C. Badcock D. R. (2011). The influence of spatial orientation on the perceived path of visual saltatory motion. Journal of Vision, 11, 5. 10.1167/11.9.5 21844167

[bibr13-20416695231191241] Khuu S. K. Kidd J. C. Phu J. Khambiye S. (2010). A cyclopean visual saltation illusion reveals perceptual grouping in three-dimensional space. Journal of Vision, 10, 26. 10.1167/10.14.26 21191136

[bibr14-20416695231191241] Lockhead G. R. Johnson R. C. Gold F. M. (1980). Saltation through the blind spot. Perception & Psychophysics, 27, 545–549. 10.3758/BF03198683 7393702

[bibr15-20416695231191241] Mather G. (1988). Temporal properties of apparent motion in subjective figures. Perception, 17(6), 729–736. 10.1068/p170729 3253676

[bibr16-20416695231191241] Ohmura H. (1981). Effects of subjective contours in stroboscopic motion. The Japanese Journal of Psychology, 52(4), 233–239. (In Japanese with an English abstract). 10.4992/jjpsy.52.233 7341824

[bibr17-20416695231191241] Ramachandran V. S. (1985). Apparent motion of subjective surfaces. Perception, 14(2), 127–134. 10.1068/p140127 4069942

[bibr18-20416695231191241] Shimojo S. (2013). Postdiction: its implications on visual awareness, hindsight, and sense of agency. Frontiers in Psychology, 5, 10.3389/fpsyg.2014.00196 PMC3978293 24744739

[bibr19-20416695231191241] Whitney D. Levi D. M. (2011). Visual crowding: A fundamental limit on conscious perception and object recognition. Trends in Cognitive Sciences, 15(4), 160–168. 10.1016/j.tics.2011.02.005 21420894 PMC3070834

